# Identification of Caffeic Acid Phenethyl Ester (CAPE) as a Potent Neurodifferentiating Natural Compound That Improves Cognitive and Physiological Functions in Animal Models of Neurodegenerative Diseases

**DOI:** 10.3389/fnagi.2020.561925

**Published:** 2020-11-10

**Authors:** Arpita Konar, Rajkumar Singh Kalra, Anupama Chaudhary, Aashika Nayak, Kanive P. Guruprasad, Kapaettu Satyamoorthy, Yoshiyuki Ishida, Keiji Terao, Sunil C. Kaul, Renu Wadhwa

**Affiliations:** ^1^AIST-INDIA DAILAB, DBT-AIST International Center for Translational and Environmental Research (DAICENTER), National Institute of Advanced Industrial Science and Technology (AIST), Tsukuba, Japan; ^2^CSIR-Institute of Genomics and Integrative Biology, New Delhi, India; ^3^DAILAB, Manipal School of Life Sciences, Manipal Academy of Higher Education (MAHE), Manipal, India; ^4^CycloChem Co., Ltd., Kobe, Japan; ^5^KAUL-Tech Co., Ltd., Tsuchiura, Japan

**Keywords:** caffeic acid phenethyl ester (CAPE), neurodegenerative disease, *Drosophila* model, mice model, neurodifferentiation, therapeutic potential

## Abstract

Cell-based screening of bioactive compounds has served as an important gateway in drug discovery. In the present report, using human neuroblastoma cells and enrolling an extensive three-step screening of 57 phytochemicals, we have identified caffeic acid phenethyl ester (CAPE) as a potent neurodifferentiating natural compound. Analyses of control and CAPE-induced neurodifferentiated cells revealed: (i) modulation of several key proteins (NF200, MAP-2, NeuN, PSD95, Tuj1, GAP43, and GFAP) involved in neurodifferentiation process; and (ii) attenuation of neuronal stemness (HOXD13, WNT3, and Msh-2) and proliferation-promoting (CDC-20, CDK-7, and BubR1) proteins. We anticipated that the neurodifferentiation potential of CAPE may be beneficial for the treatment of neurodegenerative diseases and tested it using the *Drosophila* model of Alzheimer’s disease (AD) and mice model of amnesia/loss of memory. In both models, CAPE exhibited improved disease symptoms and activation of physiological functions. Remarkably, CAPE-treated mice showed increased levels of neurotrophin-BDNF, neural progenitor marker-Nestin, and differentiation marker-NeuN, both in the cerebral cortex and hippocampus. Taken together, we demonstrate the differentiation-inducing and therapeutic potential of CAPE for neurodegenerative diseases.

## Introduction

Advancement in living standards and healthcare has significantly extended the average human lifespan globally. As a result, aging population (>60 years) has increased rapidly and expected to reach 2.1 billion counts by 2050. Given the close association between aging and brain dysfunctions, the incidences of brain pathologies are on the rise. Brain aging is accompanied with deteriorative anatomical and molecular changes coupled with increasing metabolic inefficiency that make the brain cells vulnerable to toxic insults (Konar et al., 2016). As a consequence, the cognitive capacities of the brain are severely compromised and give rise to neurodegenerative conditions, *viz*. Alzheimer’s disease (AD) and Parkinson’s disease (PD). Presently, ~30 million people are affected by neurodegenerative diseases globally, and this number is predicted to rise to 150 million by 2050 (Vanni et al., 2020). The etiology and consequences of brain pathologies are complex and involve the genetic and epigenetic changes and altered molecular signaling, connectivity, cellular morphology, and physiological behavior (Yankner et al., 2008). Therefore, despite the extensive research on neurodegenerative disorders, particularly AD, these factors have made it difficult to constitute an effective treatment regime. To date, only a few drugs, namely rivastigmine and memantine have achieved partial clinical success, primarily by alleviating the disease progression, yet constraints of the blood-brain barrier and bioavailability compromised their efficacy (Becker et al., 2008; Casey and Jones, 2010).

Neurodegenerative disorders though marked by their complex etiology and symptoms, the majority of these show atrophy of the neural connections as a prime feature. Of note, maintenance of these connections is required for brain plasticity and cognition that are indispensable for brain health (Gonzalez-Escamilla et al., 2018; Fleischer et al., 2019). Reduced neural connectivity has been observed in AD, traumatic brain injuries, cognitive aging, and malignancies, while precocious or their delayed development has been associated with autism and schizophrenia (Yizhar et al., 2011; Zalesky et al., 2015). Therefore, preserving the existing framework and reviving the lost neural architecture hold the key to therapeutics for brain disorders. The formation, maturation, and stability of neuronal connections is an orchestrated event involving multiple cellular mechanisms *viz*. neurogenesis, neurodifferentiation, synaptogenesis, and remodeling. Of note, mature neurons being nondividing cells, are difficult to revive, and therefore differentiation therapy that instigates neurite induction, extension, and guidance (Tanaka et al., 2018) in existing neurons is considered to be a valuable preventive regime for neurodegenerative disease. Such regimes offer the unique opportunity to revive the neurons with lost projections, post-injuries, neurodegenerative disorders (Jeon et al., 2019), or the brain malignancies (Guichet et al., 2013).

Among the differentiation-inducing reagents, natural compounds are more favored owing to their long-lasting holistic action with minimal adverse effects, thus enabling them to be recruited both as preventive and recovery measures. Although several natural compounds were claimed to potentiate neuronal differentiation, a comprehensive screening of these for recovery of neurodegeneration is still largely elusive. Moreover, the lack of phenotypic and molecular characterization of these compounds hampered the establishment of their therapeutic value in clinics. In this regard, cell-based differentiation assay allows high-throughput phenotype screening of compounds that can also elucidate their potential molecular activities (Alves et al., 2011). In the present study, using IMR32 human neuroblastoma cells, we performed a cell-based screening of 57 natural compounds for the differentiated phenotype to assess their potential relevance in repair and regenerative potential for neurodegenerative disease. In a three-step screening, we identified caffeic acid phenethyl ester (CAPE) as one of the potential neurodifferentiation-inducing compounds. CAPE is a natural phenolic compound, an ester of caffeic acid and phenethyl alcohol. It is a bioactive component of New Zealand honeybee propolis and causes diverse biochemical activities such as anticancer, antioxidant, anti-inflammatory, cytotoxic, and antimicrobial (Murtaza et al., 2014). Neuroprotective properties of CAPE have also been exploited in ischemic brain injuries for its anti-oxidant and anti-inflammatory properties (Wei et al., 2004; Khan et al., 2007). CAPE also suppressed diabetes-induced oxidative stress and expression of neuroinflammatory markers—tumor necrosis factor (TNF)-α and interferon (IFN)-γ (Celik and Erdogan, 2008). Caffeic acid, the principal component of CAPE also inhibited neuronal apoptosis and astrocyte proliferation, recovered brain atrophy post-neurological insults including ischemia and epilepsy in animal models (Zhang et al., 2007; Yiş et al., 2013). We had earlier characterized CAPE as a potent inhibitor of mortalin, i.e., a stress chaperone known to have the function in carcinogenesis, and thereby inhibiting its stemness and proliferative activities (Yun et al., 2017). We also showed that γ-cyclodextrin (γCD) complex with CAPE enhances its antiproliferative potency (Wadhwa et al., 2016; Ishida et al., 2018). Recently, we elucidated pro-hypoxia and anti-stress activities of CAPE (Bhargava et al., 2018b). These findings have established that CAPE is a potent bioactive compound and could instigate multiple molecular responses in treated cell/animal models. Moreover, recent findings on caffeic acid derivatives have shown its neurogenesis and neuroprotection function (Fu et al., 2013; Moosavi et al., 2017), however, its underlying cellular and molecular mechanisms remain elusive.

Hence, in the present study, we performed cellular and molecular characterization of CAPE bioactivities and revealed that it is a potent neurodifferentiating agent and modulates expression levels of neurofilament (NF)-200, microtubule-associated protein 2 (MAP2), neuronal nuclei (NeuN), postsynaptic density protein 95 (PSD95), neuron-specific class III β-tubulin (Tuj1), growth-associated protein 43/neuromodulin (GAP43), and glial fibrillary acidic protein (GFAP) neuronal markers. Of note, the neurodifferentiating activity of CAPE was not specific to any cell type or species as its treatment resulted in differentiated phenotype in human GOTO neuroblastoma, rat PC12 neuroblastoma, and C6 glioblastoma cell lines. To further assess CAPE bioactivity in neurodegenerative disease *in vivo*, we enrolled *Drosophila’s* AD model (upstream activating sequences-galactose 4, UAS-GAL4) and scopolamine (SC)-induced amnesia mouse model. Interestingly, we found that CAPE supplementation improved the mobility in Alzheimer’s (UAS-GAL4) flies. Also, it benefited a range of physiological functions including viability, stress tolerance, and fecundity in Alzheimer’s (UAS-GAL4) flies. Of note, CAPE augmented memory consolidation in SC-induced amnesic mice model, while analyses of the cerebral cortex and hippocampus brain regions of CAPE-supplemented mice showed an increase in neurotrophin brain-derived neurotrophic factor (BDNF), neural progenitor marker-Nestin, and post-mitotic differentiation marker-NeuN. Taken together, the present report endorses the differentiation-inducing activity of CAPE and elucidates that it could improve cognitive and physiological functions in animal models towards restoring the homeostatic brain function.

## Materials and Methods

### Cells Culture

IMR32, GOTO (human, neuroblastoma), PC12 (rat, pheochromocytoma/neuroblastoma), and C6 (rat, glioblastoma) cell lines were obtained from the Japanese Collection of Research Bioresources (JCRB, Tokyo, Japan). IMR32, GOTO, and C6 were cultured in modified Eagle’s medium (Wako, Tokyo, Japan) supplemented with 5% (for C6) and 10% (for IMR32 and GOTO) fetal bovine serum (FBS) and 1% antibiotics in a humidified incubator containing 5% CO_2_ at 37°C as described earlier (Kalra et al., 2015). PC12 cells were cultured in RPMI-1640 with 5% FBS concentration on above standard conditions.

### Drugs and Treatments

CAPE-γ cyclodextrin complex (CAPE-γCD) were prepared in cell culture-grade dimethylsulfoxide (DMSO) at 5 mM concentration stocks and added directly to cell culture medium to adjust the working concentrations. Retinoic acid (RA) was prepared in absolute DMSO at a 10-mM concentration stock. For rodent study, SC hydrobromide (Sigma–Aldrich, St. Louis, MO, USA) dissolved in 0.9% saline (vehicle) was administered intraperitoneally (i.p.) to mice (3 mg/kg BW) and an equal volume of saline to control animals. CAPE (10 mg/kg BW) and CAPE-γCD (12 mg/kg BW) compounds were i.p. injected 1 h post-SC hydrobromide treatment. DMSO solution (0.5%), vehicle for the compounds were i.p. administered to the control counterparts. Drugs were administered for 7 days; mice were killed; and brain regions (cerebral cortex and hippocampus) were dissected out for the gene expression studies.

### Microscopic Observations

Cell morphology of control and treated neuroblastoma/glioma cells was captured under a phase-contrast microscope (Nikon, Tokyo, Japan). Microscopic observation was carried out at 10× and 20× magnification to observe the cell phenotypes induced by DMSO, CAPE, and RA reagents over 6 weeks.

### Immunofluorescence

Neuroblastoma/glioma cells at 4 × 10^3^ were seeded on glass coverslips in a 12-well plate for 24 h. Treatments with the indicated DMSO, CAPE, and RA concentrations were given for indicated time points followed by the fixation with pre-chilled absolute methanol at reverse transcriptase (RT) for 10 min. Fixed cells were subsequently permeablized with phosphate-buffered saline (PBS)-Triton-X-100 (0.2%) for 10 min followed by blocking with 2% bovine serum albumin (BSA) for 20 min. Indicated primary antibodies (please see the details of used antibodies in Supplementary Table 1) were incubated on RT for 1 h or at 4°C overnight. Cells were subsequently incubated with Alexa Fluor-conjugated secondary antibodies (Molecular Probes, Eugene, OR, USA) and counterstained with Hoechst 33258 (Roche, Basel, Switzerland) as described earlier (Kalra et al., 2018). Immunofluorescence images were acquired under a Carl Zeiss Axioplan-2 microscope and captured with a Zeiss AxioCam HRc camera. The intensity of the acquired immunofluorescence images was quantitated by the ImageJ software that was further normalized with the respective controls and represented as % change over control.

### Immunoblotting

Control and treated cells, at 70–80% confluency, were harvested on indicated time points with trypsin-ethylenediaminetetraacetic acid (EDTA; Wako, Tokyo, Japan). The cell pellets were lysed further in RIPA buffer (Sigma–Aldrich, St. Louis, MO, USA) and quantified. Ten micrograms of protein (each sample) was resolved in sodium dodecyl sulfate (SDS)–polyacrylamide gel (PAGE) and then electroblotted at methanol-activated polyvinylidene difluoride (PVDF) membrane (Millipore) using a semidry transfer unit (ATTO, Tokyo, Japan). Immunoblotting was performed with indicated antibodies (Supplementary Table 1). PVDF membranes were probed for primary and secondary (HRP-tagged; Santa Cruz) antibodies as described earlier (Singh et al., 2014). Chemiluminescence detection was performed using enhanced chemiluminescence (ECL) prime substrate (GE Healthcare, Chicago, IL, USA). Densitometric analysis was performed with ImageJ (NIH, Bethesda, MD, USA), and quantitation of each protein in control and stressed cells was normalized with their respective β-actin level.

### Semiquantitative and Quantitative Reverse Transcriptase PCR

Total RNA from control and treated cells was extracted using the QIAGEN RNeasy kit. Two micrograms of RNA were used to synthesize cDNA using the ThermoScript^®^ Reverse Transcriptase (QIAGEN) following the manufacturer’s instructions. cDNA was then subjected to polymerase chain reaction (PCR) amplification using the transcript-specific set of primers (Supplementary Table 2) and TaKaRa Ex Taq^®^ DNA polymerase (Takara, Tokyo, Japan) as described earlier (Singh et al., 2014). The PCR amplification reactions consisted of an initial 10-min denaturation step at 95°C, followed by 34 cycles at 95°C for 45 s, 60°C for 1 min, and 72°C for 45 s and a final 10 min annealing step at 72°C. Amplified products were resolved on a 1.2% agarose gel containing ethidium bromide (0.5 μg/ml) for visualization. To analyze the Nestin, NeuN, and BDNF gene expression in mice tissue, qRT-PCR was performed using specific primer sets and conditions with extracted mRNA samples from the cerebral cortex and hippocampus for mice brain in control and treated sets. Details of Nestin, NeuN, and BDNF primers and amplification conditions are enlisted in Supplementary Table 2.

### Animal Models

#### *Drosophila* AD Model

The Oregon-K strain *Drosophila melanogaster*, *UAS-Tau R406W* strain, and *ELAV-GAL*4 strain were employed in the present study. These were cultured on wheat cream agar medium at 22°C. The flies were cultured in quarter pint glass bottles. The virgins were isolated from each strain and maintained separately for 5 days. The *UAS-Tau R406W* flies (model for AD) were crossed with the flies of the *ELAV-GAL4* strain (Phelps and Brand, 1998) to obtain F_1_ progeny. Similarly, virgin flies of the wild-type (WT) strain were also crossed.

CAPE (0.5%) was thoroughly mixed during the preparation of wheat cream agar medium. The medium was poured into the quarter pint bottles and allowed to solidify. The bottles were dried, and the moisture was removed. The male and female virgin flies of the required cross (WT and UAS-GAL4 system) were transferred into these bottles. The control flies were transferred to only the wheat cream agar media. All the experiments were conducted in duplicate. In each experiment, control and treated groups were employed in parallel. Each group consists of five replicates. For determination of toxicity, the enclosed F_1_ flies (male and female) from control and treated groups were counted. For larval crawling, the neuromuscular activity of the larvae was determined on control and test groups of the third instar larvae. An agar layer (2%) was prepared on a Petri plate, and a graph sheet was placed beneath the agar plate for convenient measurement. The larva was placed on the plate and the distance it traveled in 1 min was measured, as described earlier (Nichols et al., 2012). For rapid iterative negative geotaxis (RING) assay, 10 male flies in each treatment group were transferred into tubes separately. The tubes were marked at 8 cm from the bottom. The apparatus was firmly tapped, and the picture was captured at 3 s. The number of flies crossing the 8-cm mark was recorded. The experiments were conducted twice with three replicates each time. Further, different flies belonging to each treatment group were used (Nichols et al., 2012). For fecundity, the modified method of Bokor and Pecsenye (2000) was used. In brief, 10 flies of either sex in the F_1_ generation were crossed in tubes containing Delcour media. The flies were changed to fresh Delcour media every day for 4 days. The eggs laid were counted. For aversive phototaxic suppression (APS) assay for memory, the assay was performed following the method of Seugnet et al. (2009) with minor modification. Ten male flies of each treatment group were used. The experiment was repeated twice with two replicates for each group. The apparatus consists of two chambers, a light, and a dark chamber. A filter paper was dipped in quinine hydrochloride (1 μM concentration) and kept in the light chamber. The flies were trained for 30 s in a dark chamber one at a time and then let into the light chamber after the training. The time taken for the flies to taste the quinine was noted, and the flies were kept there for 1 min. The reading was taken for the second time with the same fly to check for memory retention and the time was noted. For the thermal tolerance test, the stress response in control and drug-treated WT and Alzheimer’s flies was tested as described (Gilchrist et al., 1997). Twenty-five male flies were transferred into tubes. The tubes were kept in a water bath with the temperature of 36°C for 60 min, and the number of knocked out flies were counted every 10 min. The flies were then carefully transferred into bottles containing media, and the number of dead flies was noted after 24 h.

#### Scopolamine-Induced Amnesic Mouse Model

Male Balb/C strain mice (10 ± 2 weeks old) from the inbred colony were used for the study. Animal handling and experiments were conducted in accordance with the guidelines of the Institutional Animal Ethical Committee, CSIR-Institute of Genomics and Integrative Biology (IGIB), New Delhi, India. The experimental protocols were also approved by the central animal ethical committee of IGIB. Balb/c male mice were taken for the 7-day experiment and blindly randomized into five groups (*n* = 9 animals/group) including control (saline, DMSO), SC, CAPE, and CAPE-γCD pre-treated groups followed by SC treatments. Concentrations of saline, DMSO, and SC were taken at 9%, 0.05%, and 3 mg/kg, respectively, of the mice body weight (BW). For CAPE and CAPE-γCD, respectively, 10 and 12 mg/kg concentrations were taken before the 3-mg/kg SC treatments in randomized mice groups. For behavior/novel object recognition (NOR) test, control and treated (CAPE and CAPE-γCD) Balb/c male mice were analyzed daily for their behavior properties and anomalies for 7 days of the experiment. NOR test was used to assess memory consolidation ability of the mice post-treatment with different combinations of drugs. Briefly, control and drug-treated animals (*n* = 9 mice per group) were habituated in the open field for two consecutive days; 5th and 6th day of drug treatment for 5 min each. On the 7th day of drug administration, mice were allowed to interact with two similar objects for 5 min after which they were returned to their home cage. After 24 h, long-term memory consolidation was assessed by replacing one familiar object with a novel one and mice were allowed to interact with the objects for 5 min. The time (%) spent with objects was calculated as TNov/(TNov + TFam) × 100 for novel and TFam/(TNov + TFam) × 100 for the familiar object. Discrimination Index (DI) for the novel objects was calculated as TNov_TFam/(TNov + TFam) where TNov is time spent with the novel object and TFam is time spent with the familiar object. The data were analyzed by ANY-maze software (Ver. 5.1 Stoelting Company, USA).

### Statistical Analyses

The Microsoft excel and Graph Pad Prism software were employed. All the experiments were performed in triplicates. Obtained data values were expressed as mean ± SEM of three individual experimental sets. Statistical analyses were executed using Student’s *t*-test or nonparametric Mann–Whitney *U* test, whichever was applicable. The data were expressed in terms of mean ± SD. Statistical significance was defined as *p*-value <0.05. The *p*-values were represented as follows: *p < 0.05, **p < 0.01, and ***p < 0.001.

## Results

### Identification of CAPE as a Potent Neurodifferentiating Compound

**Figure 1:**
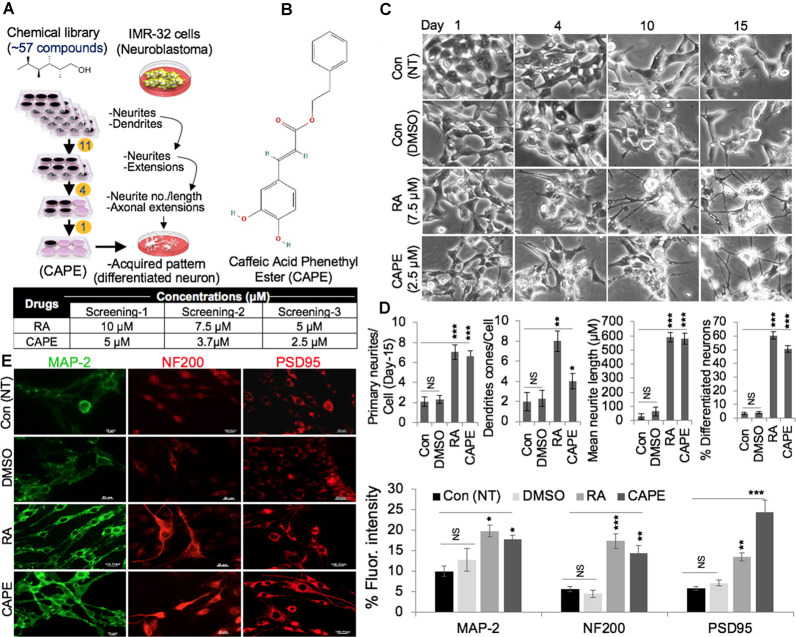
Identification of caffeic acid phenethyl ester (CAPE) as a neurodifferentiation-inducing natural compound. **(A)** Schematic diagram showing a multistep selection protocol for compounds that induced neurodifferentiation phenotype (neurites and dendrites) in IMR32 (human neuroblastoma cells). The concentration of retinoic acid (RA) and CAPE used in three screening steps are summarized in Table. **(B)** The structure of CAPE is shown. **(C)** Phase-contrast images (from days 1, 4, 10, and 15) showing differentiating phenotypes nontreated (NT, blank) and dimethylsulfoxide (DMSO)-, RA-, and CAPE-treated IMR32 cells. **(D)** Quantitation of the distinct differentiation (number of neurites, dendrites, and neurons) features in the nontreated (NT, blank) and DMSO-, RA-, and CAPE-treated IMR32 cells. **(E)** Immunostaining of MAP-2, NF200, and PSD95 in the nontreated (NT, blank) and DMSO-, RA-, and CAPE-treated IMR32 cells. Cells were treated for a week. Quantitation of immunofluorescence intensities is shown next to the images (scale bar, 20 μm). The data were expressed as mean ± SD. The *p*-values were represented as *p < 0.05, **p < 0.01, and ***p < 0.001. NS, non-significant.

We screened 57 natural compounds for their neurodifferentiating activity using human neuroblastoma (IMR32) cells. The cells were cultured in six-well dishes and subjected to the nontoxic (determined by independent cell viability assays) dose of the compounds. Cells were observed under the microscope every day for 8 days. Eleven of the 57 compounds were found to promote neurite and dendrite formations in the first round of screening. The second round of screening was conducted on 11 compounds of which four were observed to cause a strong differentiation phenotype (Figure 1A). In the third screening with four selected compounds, one compound, CAPE, caused potent neurodifferentiation of IMR32 cells, as determined by measuring of numbers and length of both neurite and dendrites (Figures 1A,B). RA was used as a positive control, while the nontreated (NT, blank) and DMSO-treated (diluent) cells were taken as internal controls. By comparative dose-dependent assays, 2.5 μM of CAPE-induced neurodifferentiation appeared to be similar to the one caused by 7.5 μM of RA (Figure 1C). Quantitative analyses of primary neurites, dendrite cones, neurite length, and percentage-differentiated neurons on day 15 affirmed that the above features in CAPE-treated IMR32 cells were largely comparable or even greater than the RA-treated cells, endorsing the potent neurodifferentiating activity of CAPE (Figure 1D); NT (blank) and DMSO-treated control cells lacked such distinct features. Furthermore, analyses of markers for mature neurons including the MAP-2, NF-200, and PSD95 showed an increase in their expression levels in response to CAPE treatment as compared with the NT (blank) or DMSO-treated control cells. Of note, an increase in the expression level of markers in CAPE-treated cells was comparable with the RA-treated cells in 1-week treated cells (Figure 1E). These data suggested that CAPE possesses potent neurodifferentiating activity.

### Molecular Analyses of CAPE-Induced Neurodifferentiation

**Figure 2:**
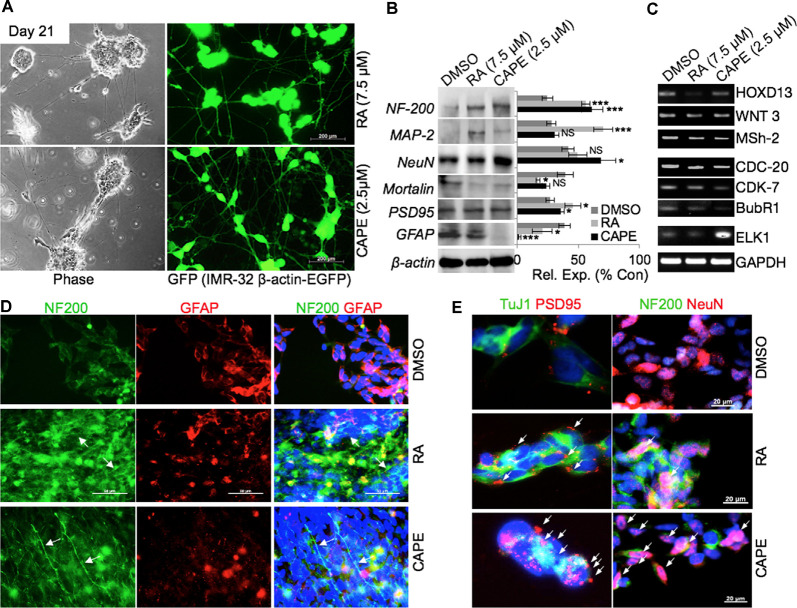
Phenotypic and molecular characterization of CAPE-induced neurodifferentiation. **(A)** Phase-contrast and fluorescent images of green fluorescent protein (GFP)-tagged IMR32 cells showing differentiated (neuron-like) cell morphology in RA- and CAPE-treated (21 days) cells (scale bar, 200 μm). **(B)** Immunoblots showing expression levels of NF200, MAP-2, NeuN, Mortalin, PSD95, and GFAP in control, RA-treated, and CAPE-treated cells. β-Actin was used as a loading control. Quantitation of their normalized expression with β-actin is shown on the right. **(C)** RNA levels as determined by reverse transcriptase (RT)-PCR of the number of genes involved in neuronal proliferation, process, and activities in control, RA-treated, and CAPE-treated cells. **(D,E)** Immunofluorescence staining showing expression and localization of NF200 and GFAP **(D)** and TuJ1 and PSD95 **(E)** differentiated neuronal markers in control, RA-treated, and CAPE-treated cells; scale bar, 50 and 20 μm, respectively. The data were expressed as mean ± SD. The *p*-values were represented as *p < 0.05, and ***p < 0.001. NS, non-significant.

In order to substantiate the above findings on the neurodifferentiation potential of CAPE, we generated green fluorescent protein (GFP)-tagged IMR32 cells and tracked their CAPE-induced differentiation by time-lapsed live cell imaging. As shown in Figure 2A, cells treated with CAPE for 3 weeks exhibited extended axonal structures. RA, an established differentiation-inducing reagent, treated cells were used as a control. Furthermore, CAPE (at 2.5 μM) induced differentiation phenotype (well-formed neuronal substructures) appeared similar to the RA-treated (7.5 μM) cells in extended 6-week differentiation time (Supplementary Figures 1, 2). In 6 weeks, CAPE-treated GFP-tagged IMR32 cells exhibited mature neuron-like features including prolonged axonal extensions, interneuronal synoptic connections, and developed dendrites, telodentria, and synaptic structures (Supplementary Figures 2A–C). Control and differentiated cells were analyzed for their protein and transcript levels. As shown in Figure 2B, there was an increase in the expression level of neuronal marker proteins namely NF-200, MAP-2, PSD95, and NeuN that mark the mature neurons, in RA- and CAPE-treated cells (Figure 2B). Of note, CAPE-treated cells showed a higher level of expression of NF-200 and NeuN as compared with the RA-treated cells. On the other hand, the level of MAP-2 remarkably enhanced in cells treated with RA, but not with CAPE (Figure 2B). GFAP (a key glial cell marker) showed a distinct decrease in response to CAPE treatment, but not in RA-treated cells (Figure 2B). These molecular changes endorsed the neurodifferentiating potential of CAPE.

**Figure 3:**
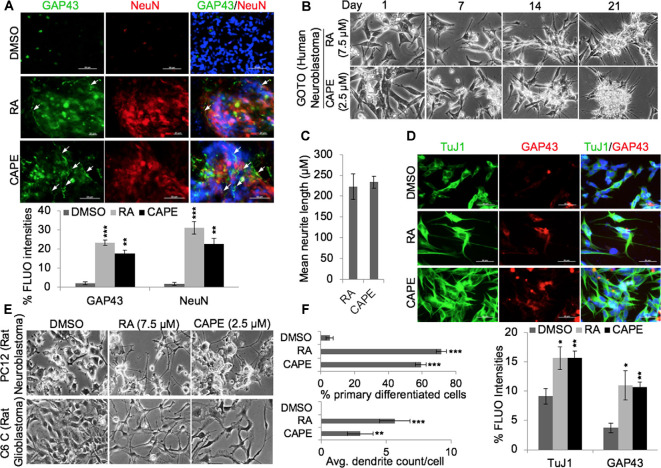
CAPE-induced differentiation was not specific to cell type. **(A)** Immunofluorescence staining showing expression and distribution of GAP43 and NeuN proteins (neuronal differentiation markers) in control, RA-treated, and CAPE-treated IMR32 human neuroblastoma cells (scale bar, 50 μm). The quantitation of their expressions is shown below. **(B)** Phase-contrast images showing differentiation phenotype in RA- and CAPE-treated human neuroblastoma cells (GOTO) derived from autonomic ganglia. **(C)** The quantitation of mean neurite length in RA- and CAPE-differentiated GOTO cells is shown. **(D)** Immunofluorescence staining showing expressions of TuJ1 and GAP43 in control, RA-differentiated, and CAPE-differentiated GOTO cells. Quantitation of immunofluorescence intensities is shown below the images (scale bar, 50 μm). **(E)** Phase-contrast images showing differentiation phenotypes post-1 week of DMSO, RA, and CAPE treatments in rat PC12 neuroblastoma and C6 glioblastoma cell lines. **(F)** Quantitation of the neurodifferentiation phenotype (percentage count of the primary differentiated neuron and average dendrite count per cell of DMSO-, RA-, and CAPE-treated rat PC12 neuroblastoma and C6 glioblastoma cells, respectively) is shown on the right. The data were expressed as mean ± SD. The *p*-values were represented as *p < 0.05, **p < 0.01, and ***p < 0.001.

Analyses of key gene expression that regulate neuronal stemness (HOXD13, WNT3 Msh-2) and proliferation [including cell cycle regulators viz. cell division cycle protein (CDC)-20, cyclin-dependent kinase (CDK)-7, BubR1] in CAPE-treated cells revealed decrease in their level of expression (Figure 2C). An increased level of ETS like (ELK)-1, a key transcription factor and modulator of epigenetic changes in differentiating neurons, was evident in CAPE-treated IMR32 neuroblastoma cells (Figure 2C). As compared with the effect of RA, CAPE-induced changes in the transcriptional levels of proliferation markers were more remarkable (Figure 2C). To further examine the neurodifferentiating activity of CAPE, co-immunostaining of NF200 (a mature neuronal marker) and GFAP (glial maker) was performed 21 days post-treatment. As shown in Figure 2D, CAPE-treated IMR32 cells exhibited increased expression and localization (along with extended axons, marked by white arrow) of NF200 (Figure 2D). While an increase in NF200 was observed in RA-treated cells, its localization across the axonal extension was indistinct (Figure 2D). Furthermore, the decrease in GFAP expression was clearly observed in CAPE-treated neuroblastoma cells as compared with their untreated counterpart. To analyze neuronal synaptic state in above-treated cells, expression analysis of PSD95, along with TuJ1, a neuron-specific class III beta-tubulin, was examined. As shown in Figure 2E, higher distribution of PSD95 (as marked by multiple red PSD95 foci, i.e., a feature of the mature neuron) on CAPE-differentiated IMR32 cells was observed as compared with the untreated control and RA-treated cells. Also, a higher level of expression of NeuN (mature neuronal nuclei) was observed in CAPE-treated IMR32 cells. These molecular changes endorsed that CAPE instigated a potent neurodifferentiating activity in IMR32 human neuroblastoma cells (Figure 2E). Of note, although RA-treated cells showed a substantially high level of expression of NF200, CAPE-treated cells showed relatively longer neurites. Quantitative analysis of fluorescence intensities of NeuN along with growth-associated protein (GAP)43, an axonal regeneration marker that expresses greatly in axonal neurites/growth cone, showed their higher levels and GAP43 localization across axonal extension/neurites (marked by white arrows) on CAPE-differentiated IMR32 cells (Figure 3A). The CAPE-induced phenotypes of differentiating neurons were apparent and comparable with the RA for its potent neurodifferentiating activity (Figures 2, 3A).

To rule out the possibility that the neurodifferentiation potential of CAPE was not specific to IMR32, we extended the analyses to other neuroblastoma and glioblastoma cells. As shown in Figure 3B, CAPE (2.5 μM) instigated differentiation in GOTO (human neuroblastoma) over 21 days (Figure 3B); RA-treated (7.5 μM) cells showed similar phenotype. Microscopic analysis of differentiated GOTO cells revealed a comparable length of neurite extensions induced by both CAPE and RA treatments (Figure 3C). Expression analyses of TuJ1 and GAP43 in GOTO cells revealed their similar increase in RA as well as CAPE-treated cells as compared with the control (Figure 3D, quantitation). We also examined CAPE activity in PC12 (rat neuroblastoma) and C6 (rat glioblastoma) cells (Figure 3E). Microscopic analyses of 3-week CAPE-treated (2.5 μM) PC12 and C6 cells exhibited differentiated phenotypes as compared with the control; RA-treated (2.5 μM) cells showed similar differentiation phenotype. Quantitative analyses revealed that >50% of CAPE- and RA-treated cells were composed of differentiated neuron populations (Figure 3F). Analysis of average number of dendrites/cells revealed that the cells treated with CAPE possessed fewer number of dendrites than the RA treatment (Figure 3F).

### CAPE Improved the Cognitive and Physiological Functions in *Drosophila* Model of AD

**Figure 4:**
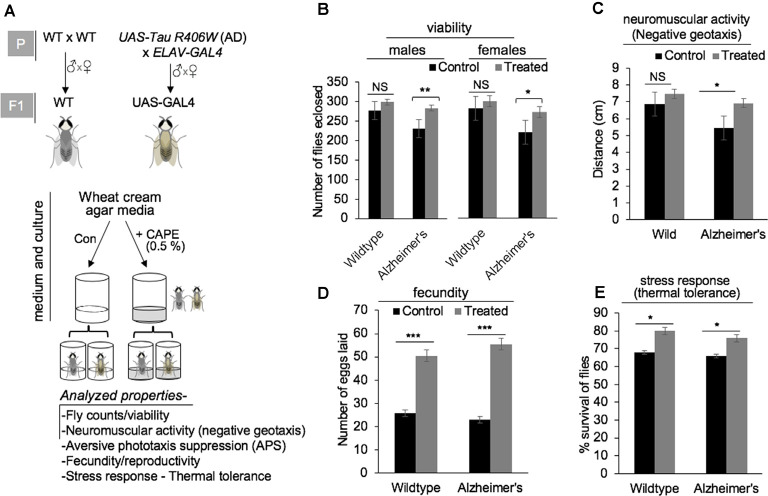
Effect of CAPE on cognitive and physiological functions in the *Drosophila* model of Alzheimer’s disease (AD). **(A)** Schematic model showing UAS-Tau R406W strain (model for AD) and ELAV-GAL4 strain at parental stage (P) and their progeny UAS-GAL4 (at F_1_), along with wild-type (WT) strains (top panel), and scheme of media/culture, CAPE treatment, and diverse investigated properties of flies obtained from all four groups (bottom panel). **(B)** Quantitation of fly counts reflecting the viability of male and female flies in wild and Alzheimer’s strains in control and CAPE-treated groups. **(C)** Quantitation showing negative geotaxic movement is increased by the treatment of CAPE in male Alzheimer’s strains. **(D)** Quantitation showing the number of eggs laid by both WT and Alzheimer’s flies were significantly increased in response to the treatment with CAPE. **(E)** Quantitation of percentage survival rate showing increased stress response/thermal tolerance in both male WT and Alzheimer’s flies in CAPE-treated groups. The data were expressed as mean ± SD. The *p*-values were represented as *p < 0.05, **p < 0.01, and ***p < 0.001. NS, non-significant.

Given the potent neurodifferentiating activity of CAPE, we next aimed to examine its effect on the WT and UAS-GAL4 [F_1_ progeny flies susceptible for AD, produced by crossing UAS-Tau R406W flies (AD model) with the ELAV-GAL4 strain flies] models of *Drosophila* (Figure 4A). The WT and UAS-GAL4 flies were cultured on wheat cream agar media supplemented with 0.5% CAPE in quarter pint bottles. The effect of CAPE was analyzed for different cognitive and physiological functions of flies including their viability, neuromuscular activity, APS, stress response, and fecundity in comparison with the respective control group (Figure 4A, below). Flies that were cultured in CAPE-supplemented media showed improved viability of male and female UAS-GAL4 (AD model) flies, whereas no significant effect was observed in WT flies (Figure 4B). Analyses of negative geotaxis movement of flies fed on CAPE-supplemented culture media revealed an improved movement of male Alzheimer’s (UAS-GAL4) strains, as compared with the WT flies (Figure 4C); yet no significant difference in WT and Alzheimer’s (UAS-GAL4) strain movement was seen with normal media (Figure 4C). Analyses of fecundity showed that the number of eggs laid by both WT and Alzheimer’s (UAS-GAL4) flies were significantly increased with CAPE supplementation (Figure 4D). Also, analyses of the stress response, an examination of percent survival rate with thermal tolerance, demonstrated an increased tolerance in both male WT and Alzheimer’s (UAS-GAL4) flies in CAPE-treated groups (Figure 4E). Analyses of phototaxic suppression (APS), and neuromuscular activity (larval crawling) in control and CAPE-treated groups yet showed no significant results in both male WT and Alzheimer’s (UAS-GAL4) flies (Supplementary Figures 3A,B). These data demonstrated that CAPE improves the cognitive and physiological functions in the *Drosophila* model of AD. The data suggest the potential of CAPE as a natural compound in the prevention of AD.

### CAPE Reversed the Deficits in Mouse Novel Object Recognition Memory With Concurrent Changes in Expression of Key Memory Markers

**Figure 5:**
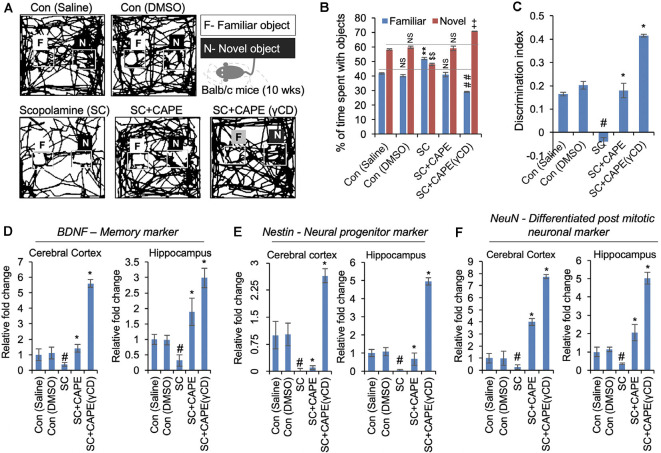
CAPE improves the cognitive behavior and memory function in scopolamine (SC)-treated mice models of neurodegenerative disease. **(A)** Readouts of novel object recognition (NOR) test showing the map of mice activities with familiar (F) and novel (N) objects in control (saline, DMSO), SC-treated, and CAPE-treated, CAPE-γCD-treated groups given treatments after SC exposure. CAPE post-treatment after SC exposure in mice was observed to improve cognitive behavior and memory function. **(B)** Quantitation showing percentage of time spent by mice with familiar and novel objects, while CAPE-γCD post-treatment found to improve mice’s attention to new objects. **(C)** Quantitation of object discriminative ability of mice showing improved ability with CAPE-γCD post-treatment in SC-treated mice. **(D–F)** Quantitative RT-PCR data showing expression of BDNF **(D)**, Nestin **(E)**, and NeuN **(F)** markers in the cerebral cortex and hippocampus regions in mice brain in control, SC, CAPE, and CAPE-γCD post-treated groups before SC treatments. Increased mRNA expression of these markers was observed in CAPE-γCD, and a bit lesser with CAPE post-treated groups after SC exposures. Histogram represents mean of the data (±SEM). Statistical analyses were performed using one-way ANOVA followed by *post hoc* student’s-Newman–Keuls test. ^#^*p* < 0.05 and *p < 0.05—significant difference as compared with saline control and SC, respectively. The data were expressed as mean ± SD. The ^##^indicated *p* < 0.01 (familiar object as compared to SC), ^$$^indicated *p* < 0.01 (novel object as compared to control-saline), and indicated ^++^*p* < 0.01 (novel object as compared to SC). NS indicated non-significant correlation.

Consistent with the tested nootropic CAPE function, improving cognitive and physiological activities in the *Drosophila* model of AD, we were prompted to examine the effect of CAPE in mice model (Supplementary Figures 4A,B). Since rodents tend to interact more with novel objects than the familiar one, we recruited their behavior to assess the memory-improving potential of CAPE using conventional NOR test (Figure 5A). As anticipated, vehicle-treated mice spent more time with the novel object (saline, 58.2%; DMSO, 59.85%) as compared with the familiar object (saline, 41.8%; DMSO, 40.14%; Figure 5B). Amnesic effect of SC was conspicuous as animals could not discriminate the novelty of the objects and spent nearly equal/less time with familiar (52%) and novel object (48%; Figure 5C). Interestingly, post-treatment with CAPE and its conjugate CAPE-γCD attenuated the object recognition memory impairment compared with the vehicle-treated control counterparts. The effect was more pronounced with CAPE-γCD (Figures 5A,B). CAPE- and CAPE-γCD-treated mice spent 58.99 and 70.78% of time with the novel object, respectively (Figures 5A,B), and significantly improved the NOR discriminatory activity (Figure 5C). The memory restoring potential of CAPE and CAPE-γCD was manifested well at the molecular level (Figures 5D–F). Memory being a complex molecular event, we examined the expression of key genes involved in different functional pathways including neurotrophic marker BDNF (Figure 5D), neuronal progenitor marker Nestin (Figure 5E), and neuronal differentiation marker NeuN (Figure 5F). Quantitative real-time PCR results revealed that SC drastically downregulated all the genes in the cerebral cortex (BDNF, 0.37-fold; Nestin, 0.02-fold; and NeuN, 0.26-fold) and hippocampus (BDNF, 0.31-fold; Nestin, 0.05-fold; and NeuN, 0.35-fold) as compared with saline control (Figures 5D–F). CAPE treatment upregulated the cortical (BDNF, 1.41-fold; Nestin, 0.1-fold; and NeuN, 4-fold) and hippocampal (BDNF, 1.89-fold; Nestin, 0.67-fold; and NeuN, 2.05-fold) expression of all genes, BDNF and NeuN being higher than vehicle group while Nestin expression was higher than SC group but lesser to vehicle control (Figures 5D–F). CAPE-γCD substantially enhanced the expression of all cortical (BDNF, 5.6-fold; Nestin, 2.64-fold; and NeuN, 7.73-fold) and hippocampal (BDNF, 2.98-fold; Nestin, 4.95-fold; and NeuN, 5.02-fold) genes as compared with vehicle control (Figures 5D–F).

## Discussion

In the present study, we have identified and characterized CAPE as a potent neurodifferentiating natural compound that improves cognitive and physiological functions in *in vivo* models. CAPE, in earlier reports, was identified to be a potent inhibitor of NF-κ B activation causing immunomodulatory and anti-inflammatory activities (Natarajan et al., 1996). Multiple reports elucidated the role of CAPE in maintaining brain function homeostasis by inhibiting cytotoxicity in cerebellar granule cells induced by low K(+) levels (Amodio et al., 2003), H_2_O_2_ led-oxidative stress (Chen et al., 2012), acrolein-induced toxicity in hippocampal cells (Huang et al., 2013), and pentylenetetrazole-induced status epilepticus (Yiş et al., 2013). We had earlier characterized CAPE as a potent inhibitor of mortalin protein and thereby inhibiting the stemness and proliferative activities (Yun et al., 2017). We further demonstrated that CAPE when complexed with γCD enhances its antiproliferative potency in *in vitro* and *in vivo* model systems (Wadhwa et al., 2016; Ishida et al., 2018). Recently, we had also elucidated that CAPE possesses pro-hypoxia and anti-stress activities (Bhargava et al., 2018b). These recent findings established that CAPE is a potent bioactive compound and instigate multiple molecular responses in cells. In the present investigation, we screened 57 compounds for their neurodifferentiating bioactivities in IMR32 neuroblastoma cells. In a three-step screening, CAPE strongly induced neurodifferentiation phenotype. Of note, CAPE promoted differentiation at a lesser (2.5 μM) concentration as compared with the RA (5 μM), a known differentiation agent. Such low requisite of CAPE may be beneficial for cells for differentiation and also drug resistance, often an outcome of exposure to high doses of drugs. Enrichment of mature neuronal markers (MAP-2, NF200, PSD95) in CAPE-treated differentiated cells endorsed its neurostimulating activities, defined by the acquisition of distinct differentiated neurons including induction of primary neurites, their elongation, and formation of dendrite cone (Figure 1). Evaluation of CAPE activities at the molecular level reaffirmed its differentiation-stimulating function as marked by elevated expression of mature neuronal (NF200, MAP-2, NeuN, PSD95) and decreased glial (GFAP) markers. Acquisition of differentiated phenotype, when exposed to CAPE, was also marked by restricted expression of genes regulating neuronal stemness (HOXD13, WNT3, Msh-2) and proliferation (CDC-20, CDK-7, BubR1). With NeuN-stained nuclei, and evident of NF200 and GAP43 localization at axonal extension in CAPE-differentiated neurons corroborated these observations. Higher expression and distribution of PSD95 foci in CAPE-differentiated cells further indicated the role of CAPE in synaptic communication and function in mature neurons (Figure 2). Whereas, PSD95 essentially functions in synaptic plasticity, and communication *via* interacting with the cytoplasmic tail of NMDA receptor and K (+) channels (Sheng and Sala, 2001), deregulation of its function impairs excitatory to the inhibitory ratio in synapses in neurons (Meyer et al., 2014). CAPE-modulated PSD95 expression/distribution in differentiated neurons underlined its potential application in restoring synaptic plasticity and preventing synaptic depression, and therefore warrant further investigation. All the CAPE activities seen as above were evident across the cell types and species, as its treatment stimulated differentiation in human (IMR32, GOTO) or rat neuroblastoma (PC12), and rat glioblastoma (C6) cell lines (Figure 3). These data clearly underlined that CAPE could serve as a reagent to stimulate differentiation in neuronal and glial cells, and therefore, emphasized its potential towards restoring the neuronal connectivity and physiology in the brain.

Assessment of CAPE activity for its effect on cognitive functions in *Drosophila’s* AD model revealed an improved movement in Alzheimer’s (UAS-GAL4) flies where it benefited a range of physiological functions including viability, stress tolerance, and fecundity (showed by the number of laid eggs) and also partially benefited the WT flies (Figure 4). Besides the differentiation-inducing activity, improved cognitive and physiological functions in CAPE-supplemented files endorsed the utility of CAPE in restoring the brain function homeostasis applications beyond re-establishing the neuronal connectivity by differentiation. Significant improvement in memory consolidation in CAPE-treated SC-induced amnesic mice model (Figure 5) further strengthens the claim that CAPE could augment cognitive and physiological functions in neurodegenerative diseases. Also, increased neurotrophin BDNF, neural progenitor marker Nestin, and post-mitotic differentiation marker NeuN in the cerebral cortex and hippocampus regions of CAPE-treated mice brain (Figure 5), marked its therapeutic implications for collective neuroenhancement. Neural stem cell proliferation and differentiation have been shown to acquire long-lasting gene expression changes that are tightly regulated by transcriptional machinery and epigenetic modifications (Feng et al., 2007). Similarly, we had earlier demonstrated that the memory impairing potential of SC is primarily attributed to epigenetic modifications and transcriptional control of memory markers like BDNF in mouse model (Singh et al., 2015; Goyal et al., 2020). Therefore, modulated transcript expressions of the BDNF, Nestin, and NeuN in SC-induced amnesic mice model affirmed impact of the SC on transcriptional control, deteriorating the cognitive behavior and memory function (Figure 5). However, CAPE was found to attenuate the above SC-induced impact on the transcriptional control and physiological outcome in these mice. Natural compounds have been asserted to modify mammalian epigenome through the regulation of DNA methylation and histone modifications (Carlos-Reyes et al., 2019). In particular, dietary polyphenols have been shown to act as DNA demethylating agents by inhibiting DNMT1-catalyzed methylation and affecting the bioavailability of methyl donors. Also, caffeic acid, an active constituent of CAPE was shown to inhibit DNMT1 and demethylated RA receptor β in cancer cells (Huang et al., 2011). It is worth mentioning here that DNMT governs neurogenesis by maintaining proliferation and suppressing the differentiation state of neural progenitor cells. Also, pharmacological DNMT inhibitors promote neurodifferentiation processes (Franco et al., 2017; Desai et al., 2019). Therefore, it is likely that CAPE promotes neurodifferentiation by inhibiting DNA methylation of molecular markers namely BDNF, MAP2, and PSD95, and thereby increasing their expression enabling progression of cells towards differentiating phenotype. The study warrants further investigation.

Earlier, Khan et al. (2018) showed that CAPE promotes functional recovery in traumatic brain injury mouse model *via* additive antioxidant activities. CAPE was shown to attenuate the progression of dementia in the nontransgenic model (ICV-STZ) of AD in rats (Kumar et al., 2017). Towards supporting the cognitive function, CAPE was also shown to diminish dopaminergic neuronal loss caused by 6-hydroxydopamine in rats (Barros Silva et al., 2013) and 1-methyl-4-phenyl-1,2,3,6-tetrahydropyridine-induced neurodegeneration (Fontanilla et al., 2011). Whereas, multiple findings corroborated its cytoprotective function against hypoxic-ischemic brain injury (Wei et al., 2004), 6-hydroxydopamine (Noelker et al., 2005), glutamate (Wei et al., 2008), ethambutol (Şahin et al., 2013), 3-nitropropionic acid (Bak et al., 2016), and cisplatin (Ferreira et al., 2019). In addition to the diverse CAPE activities supporting cognitive functions and providing neuroprotection, as we had stated earlier, elucidated its potent antitumor function essentially by mortalin protein inhibition (Yun et al., 2017), and by inducing pro-hypoxia and stress-modulating activities (Wadhwa et al., 2016; Bhargava et al., 2018a,b). These findings, along with the report suggesting that the uncontrolled cell proliferation in brain malignancies might be inhibited by inducing differentiation of nerve cells (Campos et al., 2010), essentially underlined implications of neurodifferentiation-inducing activity of CAPE in brain malignancies. Therefore, the present study endorses the neurodifferentiating activity of CAPE at the molecular level and suggested its applications for differentiation therapy in brain malignancies. The ailing neural connections are the key features in neurodegenerative disorders, while strategies restoring these networks are shown to instate brain plasticity and cognition, thereby augmenting the homeostatic brain function (Gonzalez-Escamilla et al., 2018; Fleischer et al., 2019). Therefore, it is crucial to sustain neural architecture and connectivity in the brain. Reagents potentiating the maturation and stability of neuronal connections essentially modulate the neurodifferentiation, synaptogenesis, and remodeling processes. Given the fact that the post-mitotic neuronal frameworks are difficult to revive in neurodegenerative/injured-condition, differentiation-therapy was recognized as a well-conceive regime to trigger neurite initiation in existing neurons towards evolving mature/functional neuron in the neural architecture (Tanaka et al., 2018; Jeon et al., 2019). Besides finding that CAPE possesses potent neurodifferentiating activity, improved cognitive and physiological brain function in the disease models of *Drosophila* and mice implicated its potential and promises as a natural and safe compound in the management of old-age-related deficits in memory, cognitive anomalies, and brain function.

## Data Availability Statement

All datasets presented in this study are included in the article/Supplementary Material.

## Ethics Statement

The animal study was reviewed and approved by Animal handling and experiments were conducted in accordance with the guidelines of the Institutional Animal Ethical Committee, CSIR-Institute of Genomics and Integrative Biology (IGIB), New Delhi, India.

## Author Contributions

AK, RK, RW, SK, and KS designed the study, interpreted the results, and wrote the manuscript. AK, RK, AC, AN, and KG performed the experiments and coordinated in result compilation. KS, YI, and KT helped in the interpretation of results and manuscript writing. RW and KS supervised the study. All authors contributed to the article and approved the submitted version.

## Conflict of Interest

YI and KT were employed by company CycloChem Co., Ltd.

SK is associated with KAUL-Tech Co., Ltd.

The remaining authors declare that the research was conducted in the absence of any commercial or financial relationships that could be construed as a potential conflict of interest.
