# Supplementary material for: Identification of Caffeic Acid Phenethyl Ester (CAPE) as a Potent Neurodifferentiating Natural Compound That Improves Cognitive and Physiological Functions in Animal Models of Neurodegenerative Diseases
Source: Front Aging Neurosci. 2020 Nov 10;12:561925. doi: 10.3389/fnagi.2020.561925 (PMC7685006; doi:10.3389/fnagi.2020.561925)
Supplement: Supplementary file 1 [file Table_1.docx]

| **Supplementary Table S1:** Detail of antibodies used for immunoblotting and immunostaining.   \| Antibody \| Source \| Catalog number \| \| --- \| --- \| --- \| \|  \| \| \| \| Anti-MAP-2 \| Sigma-aldrich \| M3696 (Rb) \| \| Anti-NF-200 \| Sigma-aldrich \| N4142 (Rb) \| \| Anti-PSD95 \| Thermo Scientific \| MA1-046 (Ms) \| \| Anti-NeuN \| Abcam \| ab177487 (Rb) \| \| Anti-GFAP \| Sigma-aldrich \| G9269 (Rb) \| \| Anti-Tuj1 \| Abcam \| ab18207 \| \| Anti-GAP-43 \| Santa Cruz \| sc-17790 \| \| β-Actin-HRP \| Abcam \| ab49900 \| \| Antibody produced indigenously (usage details below)- \| \| \| \| Rabbit anti-Mortalin \| Immunoblotting \| Immunofluorescence \| \| 1:10,000 \| 1:1000 \|   **Supplementary Table S2:** Details of primers used for qPCR and RT-PCR amplifications. | | |
| --- | --- | --- | --- | --- | --- | --- | --- | --- | --- | --- | --- | --- | --- | --- | --- | --- | --- | --- | --- | --- | --- | --- | --- | --- | --- | --- | --- | --- | --- | --- | --- | --- | --- | --- | --- | --- | --- | --- | --- | --- |
|  | | |
| *Primers* | *Primer/Nucleotides Sequence from 5'-3'* | *Tm* |
| BDNF  (qPCR) | FP 5′- TGCCAGAGCCCCAGGTGTGA-3′ | 60°C |
|  | RP 5′- CTGCCCTGGGCCCATTCACG-3′ |  |
| Nestin  (qPCR) | FP 5′-TCTCCAGAAGAGGAGGACCA-3′ | 60°C |
|  | RP 5′-TTCGAGAGATTCGAGGGAGA-3′ |  |
| NeuN  (qPCR) | FP 5′-CGTGTATCAGGATGGATTTTATGG-3′ | 60°C |
|  | RP 5′-CCATAACTGTCACTGTAGGCTGCT-3′ |  |
| GAPDH (qPCR) | FP 5′-TGCACCACCAACTGCTTAGC-3′ | 60°C |
|  | RP 5′-GGCATGGACTGTGGTCATGAG-3′ |  |
| WNT3  (RT-PCR) | FP 5′-CAAGATTGGCATCCAGGAGT-3′ | 60°C |
|  | RP 5′-TCCCTGGTAGCTTTGTCCAG-3′ |  |
| HOXD13  (RT-PCR) | FP 5′-TTCTGCTGCCCAACCTGACTTTGTAGTTCTG-3′ | 60°C |
|  | RP 5′-GAGCACTGCCTGCCTTTCCACTTGCCTCAGGGCAA-3′ |  |
| MSh-2  (RT-PCR) | FP 5′-GTCGGCTTCGTGCGCTTCTTT -3′ | 60°C |
|  | RP 5′-TCTCTGGCCATCAACTGCGGA -3′ |  |
| CDK-7  (RT-PCR) | FP 5′-ATTCGTGTTGTCCTGGGAGC -3′ | 60°C |
|  | RP 5′-GGCCTTGTAACGGTGGC-3′ |  |
| BUBR1  (RT-PCR) | FP 5′-ACGTTATTAGAAAGAGCTGTAT -3′ | 60°C |
|  | RP 5′-CATATCCTTTGGCTCATTGC -3′ |  |
| ELK1  RT-PCR) | FP 5′-CACGGGATGGTGGTGAATTC -3′ | 60°C |
|  | RP 5′-CTTGTGCTAATTCATGTTGGTCTTG -3′ |  |
| GAPDH  (RT-PCR) | FP 5′-TGGAAATCCCATCACCATCT-3′ | 60°C |
|  | RP 5′-TTCACACCCATGACGAACAT-3′ |  |
|  |  |  |
