# Supplementary material for: Identification of Caffeic Acid Phenethyl Ester (CAPE) as a Potent Neurodifferentiating Natural Compound That Improves Cognitive and Physiological Functions in Animal Models of Neurodegenerative Diseases
Source: Front Aging Neurosci. 2020 Nov 10;12:561925. doi: 10.3389/fnagi.2020.561925 (PMC7685006; doi:10.3389/fnagi.2020.561925)

SUPPLEMENTARY MATERIAL

Identification of Caffeic Acid Phenethyl Ester (CAPE) as A Potent Neuro-Differentiating Natural Compound that Improves Cognitive and Physiological Functions in Animal Models of Neurodegenerative Diseases

Arpita Konar[1,2#], Rajkumar Singh Kalra[1,#], Anupama Chaudhary[1], Aashika Nayak[3], Kanive P Guruprasad[3], Kapaettu Satyamoorthy[3,] Yoshiyuki Ishida[4], Keiji Terao[4], Sunil C. Kaul[1] and Renu Wadhwa[1*]*

Legends to the figures

FIGURE S1 | (A) EGFP-expressing IMR32 neuroblastoma cells showing differentiation phenotype in response to treatment with either RA or CAPE over the 6 weeks. **(B)** A CAPE-treated IMR32 cell at 6th week, showing differentiated phenotype marked by extended axonal structures, neurites, telodentria, and synopses.

FIGURE S2 | CAPE-differentiated EGFP-tagged IMR32 neuroblastoma cells exhibit neuronal sub-structures. **(A)** Differentiated neurons with their open extended axonal structures at synopsis end. **(B)** Differentiated neurons with their merged extended axonal structures at synopsis end. **(C)** Differentiated neurons showing developed dendrites, axonal extensions, telodentria, synopses, and synaptic terminals.

FIGURE S3 | (A) Quantitation showing aversive phototaxic suppression (APS) in both male wild type and Alzheimer's flies in control and CAPE-treated groups. **(B)** Quantitation showing neuromuscular activity (larval crawling) in both wild type and Alzheimer's flies in control and CAPE-treated groups.

FIGURE S4 | (A) Schematic diagram showing the experimental regime of treatments and grouping mice for behavioral and gene expression analyses. Balb/c male mice (10-12 weeks old) were taken for a 7-days experiment and divided into 5 groups (n=9 animals/group)

including control, DMSO control, Scopolamine (SC), and CAPE, CAPE (γCD) pre-treated groups followed by SC treatments. The behavioral test comprised novel object recognition and memory consolidation examinations. In gene expression analyses, expression analysis of Nestin, Neun, and BDNF; those are makers for neuronal progenitor, differentiation, and memory function respectively was performed. (**B)** Schematic diagram showing steps in novel object recognition test including the habituation (10 min, 2 days), familiarization (10 min, 1 day), and recognition (10 min, 1 day) performed on day 5th -6th, 7th and 8th -day post-drug treatments.

Supplementary Figure S1

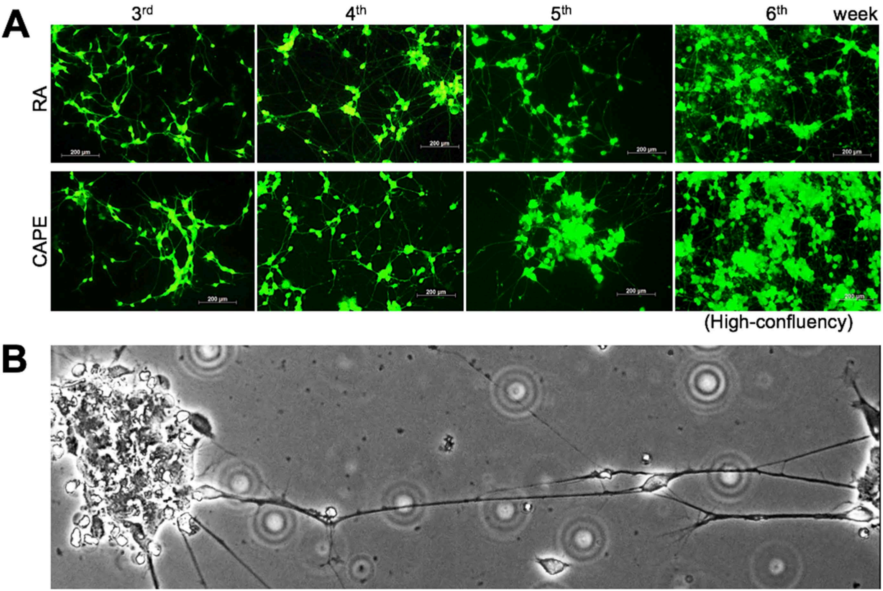

Supplementary Figure S2

A

B

C

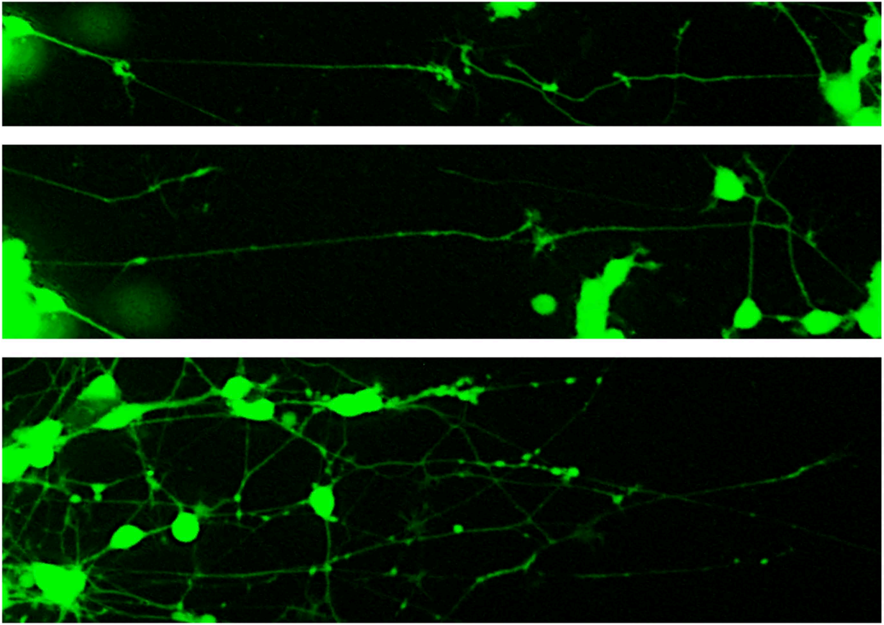

Supplementary Figure S3

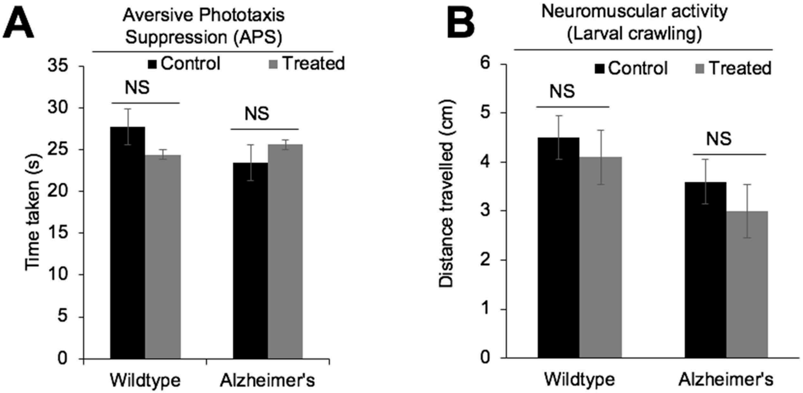

Supplementary Figure S4

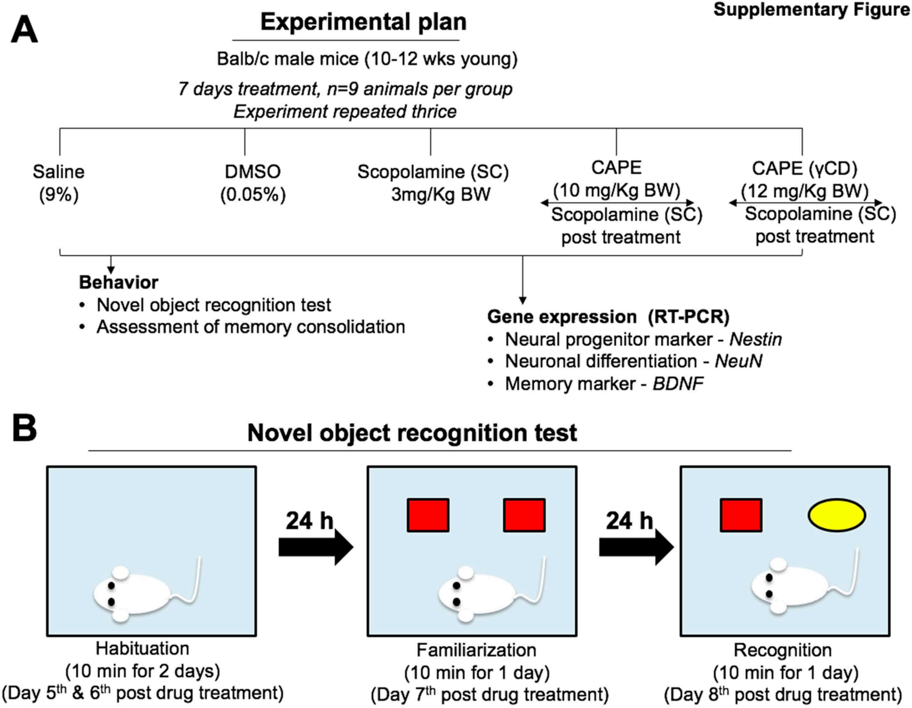

Supplement: Supplementary file 2 [file Data_Sheet_1.PDF]
